# Consumer Wearable Usage to Collect Health Data Among Adults Living in Germany: Nationwide Observational Survey Study

**DOI:** 10.2196/59199

**Published:** 2025-06-11

**Authors:** Kristin Manz, Susanne Krug, Charlotte Kühnelt, Johannes Lemcke, Ilter Öztürk, Julika Loss

**Affiliations:** 1 Department 2: Epidemiology and Health Monitoring, Robert Koch Institute, Nordufer 20, Berlin, 13353, Germany, 49 30187543567

**Keywords:** health survey, fitness tracker, smartwatch, selectivity, physical activity, sleep

## Abstract

**Background:**

The usage of consumer wearables (CWs; eg, fitness trackers and smartwatches) in the population has increased enormously within the last decade. This has resulted in a large amount of digital person-generated health data that could be used to answer vital research questions. However, little is currently known about the usage of CWs to collect health data from the population living in Germany.

**Objective:**

This study aimed to describe the ownership of consumer wearables and their usage for the collection of health data from the adult population living in Germany, as well as the motives for the collection of health data and the average wear times. In addition, this study also aimed to investigate sociodemographic and health- and behavior-related differences between the group of CW users and the group of nonusers.

**Methods:**

We used data from the nationally representative survey “German Health Update,” which was conducted through telephone interviews in 2021 and 2022. The final sample comprised 4464 adults aged 18 years and older. We derived weighted prevalences for the usage of CWs, as well as adjusted odds ratios for the ownership and the usage of CWs and their association with sociodemographic and health- and behavior-related variables.

**Results:**

Of the adult population, 19.3% (843/4459) owned a CW, of whom 77.8% (650/842) used their CW to collect health data (which corresponds to 650/4458, 15.0% of the adult population). Older people, people with a low income, and people with a lower level of physical activity (PA) were less likely to own a CW and were less likely to use it for the collection of health data. Of the CW users who collected health data, 47.2% (321/650) wore their CW during nocturnal sleep. The most frequently named motives for the collection of health data with a CW were “to observe my PA” (544/647, 85.0%), “for fun” (508/644, 79.0%), and “for support during exercising” (423/647, 66.3%). Women chose the motive “to observe my PA” and “to increase my PA” more often than men, whereas men chose the motive “to observe health issues” more often than women.

**Conclusions:**

Adults living in Germany owning a CW are younger, have a higher income, and are more physically active than individuals who do not use a CW. This means that the population groups that would be in particular need of health care are not sufficiently represented in these health datasets. Researchers should consider the selectivity of CW users when planning to use CW health data to answer research questions.

## Introduction

### Background

Individuals can nowadays collect certain health-related data with commercial wearable devices (also called “consumer wearables” [CWs]) like fitness trackers or smartwatches using integrated sensor technology (eg, accelerometers, pulse oximeters, and geolocation). Therefore, CWs can track vital parameters and behaviors, such as heart rate, physical activity (PA), or sleep [1].

The usage of CWs in the population has increased enormously within the last decade. In 2022, around 500 million of these devices were sold worldwide, which means that the sales increased by the factor 16 compared with 2014 [2]. In Germany, 7.2 million CWs were sold in 2022 [3]. However, for Germany, data from adults on the ownership of CWs and their actual use to collect health data are scarce.

### CW Data as Part of Public Health Surveillance

The prevalent usage of CWs in the population results in a large amount of digital person-generated health data that could be used to answer vital research questions or to support individual health care [4,5]. Within the public health context, these CW data could supplement the monitoring of important health behaviors, like PA and sleep, within the national public health surveillance [6,7]. Public health surveillance includes the ongoing systematic collection, analysis, and interpretation of data on the population level by, for example, using questionnaires and integrating secondary data. It aims not to give personal feedback to individuals but to identify the range and extent of population-related risk factors to facilitate goal-oriented public health responses and health policies [8].

CW data as part of public health surveillance could be used to describe the PA level of the population continuously and point out trends over time. For example, it is possible to investigate whether certain events, such as heat waves or an outbreak of a pandemic, have an effect on PA and sleep behavior.

The usage of CW data collected by individuals with their own CWs produces a variety of benefits for the research community, like no expenditures on devices and data collection as well as the availability of objective data, which are often superior to self-reports [7,9]. Furthermore, the data are collected continuously by the CW users, which enables researchers to perform retrospective analyses and to observe health behavior over a longer period of time [7]. In addition, the effort required of the participants is low, as they only have to share their data via a smartphone app.

At the same time, there are challenges in using such data for research, such as privacy and security issues, technical issues and a lack of access to the raw data [9]. The algorithms used by the companies of the CWs to convert the raw data into, for example, steps or the duration of PA at different intensities, are often not disclosed [9]. This limits the comparability of CW data over time, as the algorithm may have changed, and between different devices. In addition, a further challenge is the selectivity of the population providing CW data [10,11]. Selectivity of study samples already exists in questionnaire-based observational studies (eg, lower response rates of people with a low educational level) and may increase for data collection using respondents’ own CW [12]. A national survey of the US population showed that adults using a CW to monitor their health are younger, healthier, wealthier, and more educated than the general adult population [10]. The low representativeness of older adults using CWs was confirmed by a study from the United Kingdom based on nationwide datasets [13]. In addition, the authors of the United Kingdom study showed that CW users had a higher PA level than the general population. To the best of our knowledge, no study has so far investigated the usage of CWs in the population of Germany and characterized the user group.

Uncritical usage of CW data might lead to biased results that cannot be generalized to the whole population. Within this context, Ibrahim and colleagues [14] used the term “health data poverty” to describe the underrepresentation of certain groups in health datasets, which can foster health inequalities and create a digital health divide.

The first step to counteract health data poverty is to raise awareness of the existing inequalities in health datasets [14]. Therefore, knowledge about the characteristics of the group of CW users is important to be able to make informed decisions about the possibilities and limitations of such datasets and the interpretation of the results. Furthermore, the rapid development of the CW market and the increasing number of cheaper CWs will constantly change the user group, requiring regular characterization of the group of CW users.

### Objective

The aim of this study is to describe the CW ownership and usage among adults living in Germany based on data from a nationally representative survey. The intentions are (1) to identify CW owners and users who collect health data and their sociodemographic and health-related characteristics as compared with the population not owning and not using a CW and (2) to gain insights into the motivations of individuals to use their CW to collect health data and their user behavior (eg, the wear time of the CW per day).

## Methods

### Study Design

The assessment of CW usage was integrated into the representative population-wide survey “German Health Update” (GEDA) 2021 and 2022. GEDA is a regularly conducted cross-sectional survey with the aim of describing the respondents’ health status and health behavior and their influencing factors [12].

GEDA 2021 was conducted from July to December 2021 (n=5030) and GEDA 2022 from February 2022 to mid-January 2023 (n=33,149). In both surveys, telephone interviews were carried out using a programmed, structured questionnaire (computer-assisted telephone interview). The sampling is based on a random sample of mobile and landline telephone numbers (dual-frame method) [15]. The population comprised the German-speaking population aged 16 and older (until December 2021; after February 2022: 18 y and older), living in private households, and having a usual place of residence at the time of data collection in Germany. Measures have been taken to increase the response rate of people who are less likely to participate (eg, people with a low educational level and older people), such as oversampling.

The present analysis is limited to persons aged 18 years and older. The topic “consumer wearable usage” was assessed in GEDA in the periods from the end of October to the end of December 2021 (n=1986) and the beginning of February to mid-June 2022 (n=2478; total sample n=4464).

### Ethical Considerations

GEDA 2021 and 2022 are subject to strict compliance with the data protection provisions set out in the EU General Data Protection Regulation and the Federal Data Protection Act. The Ethics Committee of the Charité – Universitätsmedizin Berlin assessed the ethics of the study and approved its implementation (application number EA2/201/21). Participation in the study was voluntary. The participants were informed about the aims and contents of the study and about data protection. Informed consent was obtained verbally. Participant confidentiality was maintained by pseudonymizing data and presenting results in an aggregated format. No compensation or incentives were given for participating in the telephone interview.

### Measures

#### Consumer Wearable–Related Variables

The questions regarding the usage of CWs were prefaced by short definitions of the terms “wearable,” “fitness tracker” and “smartwatch.” The ownership and the usage of a CW were assessed with the questions “Do you own a wearable?” (answer categories: yes or no) and “Do you currently use your wearable to measure your PA, fitness or other health data such as blood pressure or pulse?” (answer categories: yes or no).

The usage of CWs was assessed via the questions “Do you measure your PA, fitness or other health data such as blood pressure or pulse with a fitness tracker and fitness bracelet?” (answer categories: yes or no), followed by the question “On how many days per week do you wear the fitness tracker or fitness bracelet for a total of at least 8 hours per day?” (answer options: the number of days from 1 to 7 and “no day per week”). The same questions were asked to obtain information about the usage of other types of devices (we used the terms “smartwatch” and “other device” instead of “fitness tracker or bracelet”).

The usage of a CW during nocturnal sleep was assessed with the question “Do you wear any of your devices while sleeping at night?” and the answer categories were “no,” “yes, fewer than 4 nights per week” and “yes, at least 4 nights per week.” The motives for CW usage were assessed via the question “Which of the following statements apply to you? I measure my PA, fitness or other health data with a wearable or app on my smartphone...,” and the following motives could be chosen (multiple responses were possible): “to observe how active or inactive I am,” “for support during sports activities or training,” “to motivate myself to be more physically active,” “to monitor a health problem, for example blood pressure, pulse,” “to lose weight,” “to exchange or compare myself with others,” “to eat and drink more healthily,” “because it’s fun for me,” and “for other reasons”

#### Explanatory Variables

Gender identity was used to describe gender differences. The participants were able to indicate the gender to which they felt they belonged [16]. In the current study sample, 53.8% (2395/4454) identified themselves as female (Table 1). In the analyses by gender, individuals with a different gender identity or no indication are not shown (n=10).

Participants’ age (in years) resulted from their date of birth and the date of the survey. For the analyses, age was categorized into 7 groups: 18 to 29 years, 30 to 39 years, 40 to 49 years, 50 to 59 years, 60 to 69 years, 70 to 79 years and 80 years and older.

Education was categorized into low International Standard Classification of Education (ISCED 1‐2), medium (ISCED 3‐4) and high (ISCED 5‐8) groups based on the educational and vocational qualifications of the study participants according to the 2011 version of the (ISCED 2011) [17].

Based on the self-reported monthly net income of the study participants’ households, the net equivalent income was calculated using the equivalence scale of the Organization for Economic Cooperation and Development. Missing income information was imputed using regression analysis procedures. Income groups were categorized as low (quintile 1), medium (quintiles 2‐4), and high (quintile 5) for analyses.

For the evaluation of the participants’ subjective health status, they were asked, “How would you describe your general health status?” The five response categories were summarized in the categories “very good or good” and “medium or poor” (including fair, poor and very poor).

The achievement of the endurance-related part of the World Health Organization (WHO) recommendation for PA (at least 150 min moderate to vigorous PA per week [18]) was assessed via the German version of the European Health Interview Survey – PA Questionnaire [19]. The indicator considers the weekly duration of leisure time PA and weekly cycling for transportation. The recommendation was achieved if the sum of the described activities was at least 150 minutes per week.

**Table 1.** Description of the study sample (n=4464).

		Proportion unweighted, n (%)	Proportion weighted, n (%)	Missing values
**Gender**				10
	Male	2059 (46.2)	2059 (48.8)	
	Female	2395 (53.8)	2395 (51.2)	
**Age group (years)**				0
	18‐29	322 (7.2)	322 (16.1)	
	30‐39	418 (9.4)	418 (15.7)	
	40‐49	551 (12.3)	551 (14.6)	
	50‐59	920 (20.6)	920 (19.2)	
	60‐69	1067 (23.9)	1067 (15.3)	
	70‐79	704 (15.8)	704 (10.7)	
	80+	482 (10.8)	482 (8.5)	
**Educational level**				14
	Low	237 (5.3)	237 (17.6)	
	Medium	1904 (42.8)	1904 (56.8)	
	High	2309 (51.9)	2309 (25.6)	
**Income**				0
	Low	509 (11.4)	509 (18.4)	
	Medium	2599 (58.2)	2599 (60.5)	
	High	1356 (30.4)	1356 (21.1)	

### Statistical Analysis

All the analyses were performed using a weighting factor that corrects for deviations of the sample from the population structure. First, a design weighting was carried out for the different selection probabilities (mobile and fixed networks), and then an adjustment was made to the official population figures with regard to age, gender, federal state, and district type (as of December 31, 2020) and in relation to education (Microcensus 2018). The analyses were carried out with Stata 17.0 (StataCorp LLC) using the survey procedures. Prevalences are reported with the corresponding 95% CI to display the range in which the true value falls with 95% probability (95% CIs are reported directly in the text or in the corresponding tables or figures). In bivariate analyses, differences between groups were determined using Pearson *χ*^2^ tests. In addition, multivariable adjusted odds ratios (ORs) with corresponding 95% CIs were calculated using logistic regression analyses to identify relevant associations between the sociodemographic and health-related variables and the CW-related outcome variables (“ownership of a CW” and “collection of health data”). In the regression analysis about the “ownership of a CW” 82 participants (82/4464, 1.8% of the total sample) had to be excluded due to missing values for the included variables, and 83 participants had to be excluded (83/4464, 1.9% of the total sample) from the regression analysis regarding the “collection of health data.” Following the recommendations of Statistics Canada, estimations with a coefficient of variation of 16.5% to 33.5% were flagged as having a high degree of uncertainty (estimations with even higher coefficients of variations would not be released; this does not apply to any of our results) [20]. A statistically significant difference between groups was assumed if the corresponding *P* value was less than .05.

## Results

### Study Sample

The study sample comprises 4464 participants aged 18 years and older. The weighted and unweighted distributions of the sample across gender, age, educational, and income groups are presented in Table 1. Participants younger than 40 years of age and those with a low educational level are underrepresented within the study sample, which is corrected by the weighting factor used.

### Ownership of a CW

Among the adult population, 19.3% (843/4459) owned a CW (Table 2). In the bivariate analyses, age, educational level, income, subjective health status and PA behavior were significantly associated with CW ownership (Table 2).

**Table 2.** Proportion of consumer wearable ownership stratified by sociodemographic and health- and behavior-related variables.

		Ownership of a CW^a^					
		n	Yes, % (95% CI)	n	No, % (95% CI)	n	*P* value^b^
Total		4459	19.3 (17.6‐21.1)	843	80.7 (78.9‐82.4)	3616	—^c^
**Gender**		4449					.99
	Female		19.4 (17.0‐22.1)	421	80.6 (77.9‐83.0)	1970	
	Male		19.4 (16.9‐22.1)	422	80.6 (77.9‐83.1)	1636	
**Age group (years)**		4459					
	18‐29		20 (15‐26)	74	79.7 (73.8‐84.6)	248	
	30‐39		31.2 (25.5‐37.6)	129	68.8 (62.4‐74.5)	289	
	40‐49		30.0 (24.8‐35.9)	163	70.0 (64.1‐75.2)	388	
	50‐59		20.1 (16.8‐23.8)	222	79.9 (76.2‐83.2)	696	
	60‐69		13.0 (10.3‐16.3)	177	87.0 (83.7‐89.7)	888	
	70‐79		7 (4-10)	61	93.5 (90.5‐95.6)	643	
	80 years and older		2 (1-4)^d^	17	97.6 (95.6‐98.7)	464	
**Educational level**		4447					<.001
	Low		9 (6-15) ^d^	22	90.6 (85.2‐94.1)	215	
	Medium		20.3 (17.8‐23.0)	334	79.7 (77.0‐82.2)	1568	
	High		23.6 (21.2‐26.2)	486	76.4 (73.8‐78.8)	1822	
**Income**		4459					<.001
	Low		11 (8‐15)	63	89.0 (84.9‐92.1)	443	
	Medium		18.6 (16.4‐21.1)	444	81.4 (78.9‐83.6)	2153	
	High		28.4 (24.7‐32.5)	336	71.6 (67.5‐75.3)	1020	
**Subjective health status**		4459					<.001
	Good or very good		22.0 (19.9‐24.3)	695	78.0 (75.7‐80.1)	2616	
	Medium or poor		11.6 (9.1‐14.6)	148	88.4 (85.4‐90.9)	1000	
**PA**^e^ **as recommended**^f^		4402					<.001
	Yes		23.3 (20.6‐26.1)	511	76.7 (73.9‐79.4)	1774	
	No		15.4 (13.2‐17.9)	325	84.6 (82.1‐86.8)	1792	

a CW: consumer wearable.

b Pearson *χ*2 test

c Not applicable.

d Estimation with high uncertainty due to a small sample size.

e PA: physical activity.

f At least 150 min PA per week (WHO recommendation).

**Figure 1.**
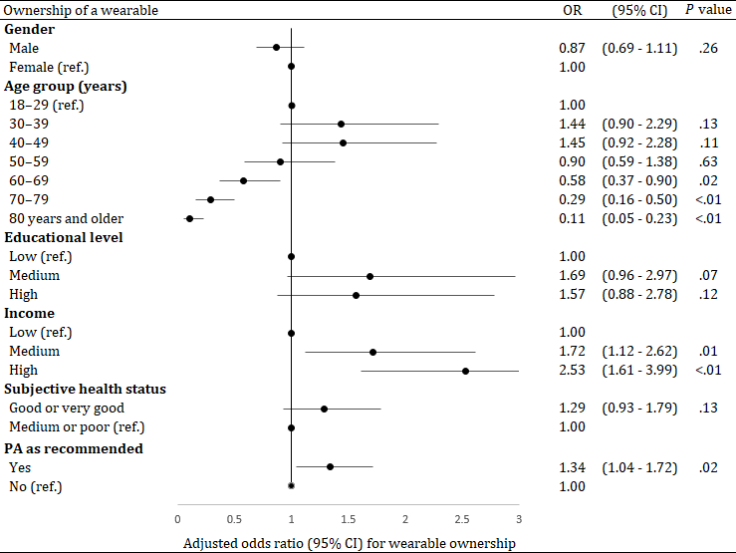
Results of a multivariable logistic regression analysis (dependent variable: “Ownership of a consumer wearable,” categories: yes or no [reference], adjusted OR with 95% CI, n=4382). “PA as recommended” refers to at least 150 minutes of PA per week (WHO recommendation). OR: odds ratio; PA: physical activity; ref: reference category; WHO: World Health Organization.

The multivariable regression analysis, including the dependent variables gender, age, education, income, subjective health status and PA level, revealed that the ownership of a CW was significantly associated with the age, income and PA level of the participants (Figure 1). Adults aged 60 years and older were less likely to own a CW than 18- to 29-year-olds: In 60- to 69-year-olds, the odds of owning a CW was 0.6 times lower than of those aged 18 to 29-year (*P*=.02), in 70- to 79-year-olds the odds was 0.3 times lower (*P*<.01), and of those aged 80 years and older the odds was 0.1 times lower (*P*<.01). Adults with a medium income had 1.7 greater odds to own a CW than adults with a low income (*P*=.01) and adults with a high income had 2.5 greater odds (*P*<.01). In addition, adults who achieved the WHO recommendation for PA had 1.3 greater odds to own a CW than adults not achieving the WHO recommendation (*P*=.02).

### Usage of a CW to Collect Health Data

Of the adult population, 15.0% (650/4458) used a CW to collect health data (Table 3). This corresponds to 77.8% (650/842) of the CW owners. In the bivariate analyses, we compared CW users with nonusers, and the respondents’ age, education, income, subjective health status, and PA level were significantly associated with the collection of health data with their own CW (Table 3).

**Figure 2.**
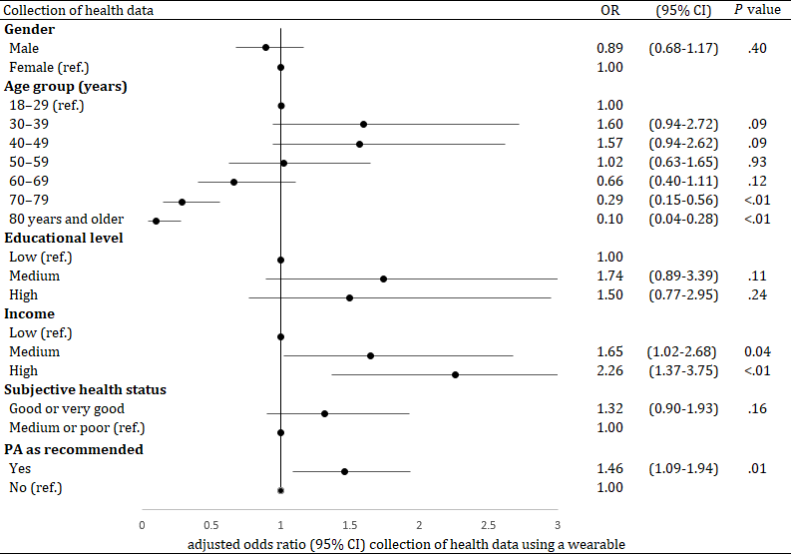
Results of a multivariable logistic regression analysis (dependent variable: “Collection of health data using a consumer wearable,” categories: yes or no (reference), adjusted OR with 95% CI, n=4381). “PA as recommended” refers to at least 150 minutes of PA per week (WHO recommendation). OR: odds ratio; PA: physical activity; ref: reference category; WHO: World Health Organization.

In the multivariable regression analysis, the variables age, income and PA level remained significant predictors for the collection of health data (Figure 2). The odds of adults aged 70 to 79 years to collect health data using a CW was 0.3 times lower than in 18- to 29-year-olds (*P*<.01). Participants aged 80 years and older had 0.1 lower odds to collect health data using a CW than the youngest age group (*P*<.01). In addition, participants who achieved the WHO recommendation for PA had 1.5 higher odds to collect health data than participants who did not achieve this recommendation (*P*=.01).

**Table 3.** Proportion of collection of health data using respondents’ own consumer wearable stratified by sociodemographic and health- and behavior-related variables.

		Collection of health data using respondents’ own CW^a^					
		n	Yes, % (95% CI)	n	No, % (95% CI)	n	*P* value^b^
Total		4458	15.0 (13.4‐16.7)	650	85.0 (83.3‐86.6)	3808	—^c^
**Gender**		4448					.99
	Female		15.1 (12.9‐17.6)	322	84.9 (82.4‐87.1)	2068	
	Male		15.1 (12.9‐17.7)	328	84.9 (82.3‐87.1)	1730	
**Age group (years)**		4458					<.001
	18‐29		15 (1‐21)	53	85.0 (79.5‐89.3)	269	
	30‐39		25 (20‐32)	96	74.7 (68.4‐80.0)	322	
	40‐49		23.6 (18.8‐29.1)	130	76.4 (70.9‐81.2)	420	
	50‐59		15.8 (12.9‐19.1)	177	84.2 (80.9‐87.1)	741	
	60‐69		10.3 (7.8‐13.4)	139	89.7 (86.6‐92.2)	926	
	70‐79		4 (3-7)^d^	45	95.6 (92.9‐97.3)	659	
	80 years and older		2 (1-4)^d^	10	98.5 (96.5‐99.4)	471	
**Educational level**		4446					.01
	Low		7 (4-12)^d^	14	93.0 (87.8‐96.1)	223	
	Medium		16.1 (13.8‐18.6)	260	83.9 (81.4‐86.2)	1642	
	High		17.9 (15.7‐20.2)	375	82.1 (79.8‐84.3)	1932	
**Income**		4458					<.001
	Low		9 (6-12)	46	91.4 (87.6‐94.2)	460	
	Medium		14.7 (12.6‐17.0)	342	85.3 (83.0‐87.4)	2255	
	High		21.6 (18.3‐25.2)	262	78.4 (74.8‐81.7)	1093	
**Subjective health status**		4458					<.001
	Good or very good		17.3 (15.3‐19.4)	541	82.7 (80.6‐84.7)	2769	
	Medium or poor		8.6 (6.5‐11.4)	109	91.4 (88.6‐93.5)	1039	
**PA**^e^ **as recommended**^f^		4401					<.001
	Yes		18.7 (16.3‐21.4)	407	81.3 (78.6‐83.7)	1878	
	No		11.4 (9.4‐13.6)	237	88.6 (86.4‐90.6)	1879	

a CW: consumer wearable.

b Pearson *χ*2 test.

c Not applicable.

d Estimation with high uncertainty due to a small sample size.

e PA: physical activity.

f At least 150 min PA per week (WHO recommendation).

### User Behavior

The most commonly used CW to collect health data was a smartwatch. A total of 74.5% (456/648, 95% CI 69.1%‐79.3%) of the CW users who collected health data used a smartwatch, of whom 71.8% (341/456, 95% CI 65.0%‐77.8%) wore their smartwatch for at least 8 hours per day. Fitness trackers were used by 60.7% (414/650, 95% CI 54.7%‐66.4%), of whom 70.7% (310/413, 95% CI 63.0%‐77.3%) wore it for at least 8 hour per day. Furthermore, 26.8% (214/650, 95% CI 22.1%‐32.2%) used a CW other than a smartwatch or fitness tracker. The results show that some of the participants used more than one device.

Of the CW users who collected health data, 47.2% (321/650, 95% CI 41.4%‐53.2%) wore their CW during nocturnal sleep for at least 4 nights per week, and an additional 6% (42/650, 95% CI 4.0%‐9.1%) wore it for fewer than 4 nights per week. Consequently, 46.7% (287/650, 95% CI 40.8%‐52.7%) did not collect sleep data.

### Motives for CW Usage

**Figure 3.**
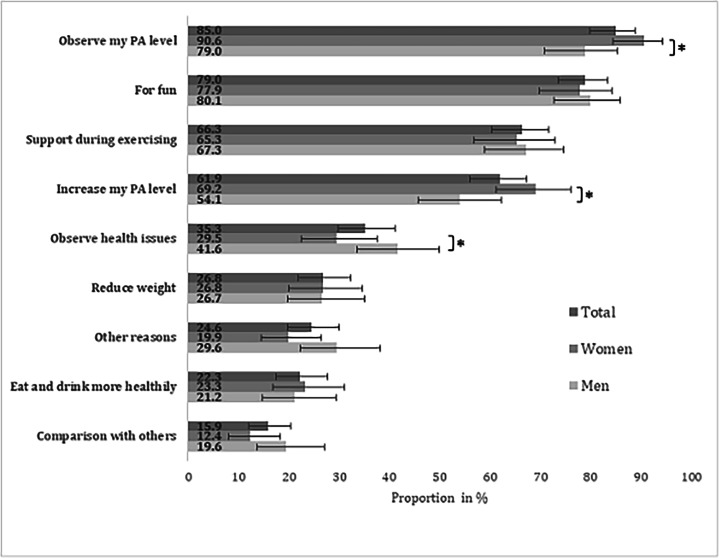
Reported motives for the usage of consumer wearables (multiple responses were possible). Proportions with 95% CIs are shown. Sample: wearable users who collect health data (n=650). PA: physical activity. *P<.05.

The most frequently chosen motives for the usage of a CW were “to observe my PA level” (544/647, 85.0%), “for fun” (508/644, 79.0%), “for support during exercise” (423/647, 66.3%), and “to increase my PA level” (386/647, 61.9%). Women named the motives “to observe my PA level” and “to increase my PA level” more often than men (287/321, 90.6% vs 257/326, 79.0%, *P*=.01; 211/321, 69.2% vs 175/326, 54.1%, *P*=.01, respectively), whereas men chose the motive “to observe health issues” more often than women (137/326, 41.6% vs 103/321, 29.5%, *P*=.04). The described differences between women and men remained statistically significant when the analyses were adjusted for age, educational level, income, subjective health status and PA level (results of multivariable logistic regression analyses, data not shown). The motives for the usage of a CW for the total sample and stratified by gender are shown in Figure 3.

## Discussion

### Principal Findings

To our knowledge, this is the first nationwide study to describe comprehensively the ownership of CWs and the usage of CWs for the collection of health data among the adult population living in Germany. In addition, the selectivity of the user group was investigated. We found that almost one-fifth of the adult population living in Germany owned a CW, 77.8% (650/842) of whom used their CW to collect health data (which corresponds to 650/4458, 15.0% of the adult population). Older people, people with a low income, and people with a lower PA level were less likely to own a CW and to use their own CW to collect health data. Of the 4 most frequently chosen motives for collecting health data using a CW, 3 were related to PA behavior, but using the CW for fun was also a common motive.

### Comparison With Previous Work

Compared with our results, a nationwide survey from Canada conducted in 2017 showed a slightly higher prevalence of CW ownership, whereas the prevalence of using the device for the collection of health data in relation to the total population was almost the same: 25% of the Canadian population owned a CW (843/4459, 19% in this study) and 57% of the owners used the device to collect health data [21] (which corresponds to 14% of the population; 15% (650/4458) in our study). A nationwide study from the United Kingdom conducted in 2018 assessed the usage of a CW to collect health data and revealed similar prevalences to those of this study, with 14% using a smartwatch or fitness tracker [13], whereas a nationwide survey from the United States conducted in 2019 revealed a higher prevalence, with 30% using a CW in the past 12 months [10]. However, it should be borne in mind that the data collection for the studies described above took place 3 to 5 years before our study and that the prevalences of the ownership and usage of CWs may have increased in the meantime.

Our results concerning the selectivity of the group of CW owners and users confirm the findings of studies from the United States [10], the United Kingdom [13] and Canada [21] for the population living in Germany. Among CW users, people who are the most in need of health care and health promotion, such as older people and socio-economically disadvantaged people, are underrepresented. Older people (aged 60 y and older) may use CWs less frequently because they are less tech savvy, they may be more reluctant to use new technologies and they may have less fine motor skills or impaired vision, making it difficult to operate a CW or smartphone [22,23]. A study from the United States showed that seniors with higher technical self-efficacy were more likely to use a CW [22]. Thus, the improvement of technical skills in combination with the further tailoring of devices to the needs of older people could increase the user behavior in this age group.

The lower likelihood of people with low income owning and using a CW in this study is consistent with the findings from the United States and Canada [10,21]. Furthermore, in the nationwide study from Canada, it was shown that costs are a common mentioned reason for not owning a CW [21]. However, due to the increasing proportion of low-cost CWs on the market, the economic aspect will probably become less important in the future. In addition, adults with a lower income often have a poorer state of health and lower PA levels, which might be accompanied by less interest in these behaviors and thus less interest in tracking health and PA indicators using a CW [24,25].

Our results revealed that adults with a higher PA level were more likely to own a CW and to use it for the collection of health data. A higher level of PA in CW users than in nonusers has also been shown in previous observational studies [26,27]. People with a higher level of PA may have a greater interest in their PA behavior and are thus more likely to track it using a CW than people with a lower level of PA [28]. This could also explain our findings on the motives for using a CW: 3 of the 4 most frequently named motives were related to PA behavior. Conversely, the use of CWs can influence the PA behavior of the users and increase their PA level. The authors of an umbrella review concluded that the usage of CWs is effective in increasing PA [29]. However, the positive effects are significantly smaller when the usage of a CW is the only measure compared with multifaceted interventions to promote PA [30]. In addition, little is known about the long-term effects of CW usage on PA behavior.

Health data collected by the population using their own CW can be used for different purposes. Our results indicate that almost 20% (843/4459) of the members of the adult population living in Germany who use a CW and collect health data wear the device regularly during daytime and thus provide data that reflect the general behavior of this group. However, only half of the CW users wear their device during nocturnal sleep, which restricts the possibilities for sleep analyses significantly.

### Strengths and Limitations

A strength of our study is that we used a nationally representative survey to investigate the ownership and usage of CWs and the motives in the adult population living in Germany. In addition, we analyzed a variety of sociodemographic and health- and behavior-related predictors of CW ownership and usage. Our results can be used to inform researchers designing research projects including CW data, as well as practitioners. Nevertheless, the following limitations of our study must be considered. First, the analyses of the factors predicting the ownership and usage of CWs might miss predictors of CW ownership or usage because they were not assessed in our study. For example, further societal indicators, like cultural background, might have an impact on CW usage as well as the technical competence of a person. Second, our results provide some initial insights into the motives of using CWs, but we might have missed information about relevant motives because almost one-quarter of the sample used the answer category “other reasons.” Further research using open-ended questions or qualitative research approaches could help to better understand the motives for CW use. Third, small sample sizes of specific subgroups, such as younger adults and adults with a low educational level, resulted in estimations with higher measurement uncertainty. We have marked results with high measurement uncertainty to make this limitation transparent. In addition, we were not able to perform more in-depth analyses, such as further subgroup analyses, due to the small sample size in the subgroups. Fourth, the assessment of data using self-reports is a limitation because we cannot rule out the possibility of biased results due to recall bias or different interpretations of terms like “wearable,” “smartwatch,” or “fitness tracker.” However, we included short definitions of the aforementioned terms in the interviews to minimize the possibility of different interpretations. Finally, the cross-sectional design of the dataset does not allow us to clarify predictive directions. Future longitudinal study designs are needed to contribute to the understanding of the long-term effects of CW use on PA behavior.

### Implications for Research and Practice

In our opinion, a major challenge when using health data collected by individuals through their own CW is that the sample is selective and does not represent the general population. Consequently, parts of the population who are particularly likely to need health care and the improvement of the health care system, such as older and socially disadvantaged people, would not benefit from these research approaches or might even be harmed. Thus, the use of data collected with respondents’ own device is, according to the current status, not suitable for the surveillance of health behavior on the populational level. A possibility to reduce the selectivity could be to supplement the sample with a randomly chosen sample of individuals who do not own a CW. This group could be sent a fitness tracker for the period of the study. Oversampling of difficult-to-reach groups of the population, like older people or people with low incomes, as well as a study design and study information adapted to difficult-to-reach target groups can further reduce selectivity.

However, health data collected by individuals using their own CW could be used to achieve other research aims than population-wide surveillance of health behavior. For example, they could be used to describe trends in PA and sleep behavior and to investigate whether certain events, like heat waves, have an impact on these behavior. In addition, these data could be used to describe individual trends in PA behavior in the form of longitudinal analyses to, for example, evaluate how the transition from employment to retirement affects PA and sleep behavior.

Researchers and practitioners should still consider carefully which parts of the population are underrepresented in their CW datasets and which conclusions can be drawn, and they should communicate the selectivity of the sample transparently. Eventually, it depends on the research question whether CW data collected by individuals with their own device are suitable for answering it. The results of our study might support the decision process that researchers should execute when planning a study using CW data.

### Conclusion

Our results indicate that one-fifth of the adult population living in Germany owns a CW. Individuals owning a CW are younger, have higher incomes, and are more physically active than the general population. The usage of such data describing the health behavior at the population level will therefore overestimate health-promoting behaviors such as PA.

## Abbreviations

CW: consumer wearable
GEDA: German Health Update
ISCED: International Standard Classification of Education
OR: odds ratio
PA: physical activity
WHO: World Health Organization
